# Prevention is better than cure: effects of errors on memory performance during spatial learning in healthy aging

**DOI:** 10.1007/s40520-020-01603-2

**Published:** 2020-05-30

**Authors:** Inge Scheper, Inti A. Brazil, Ellen R. A. de Bruijn, Larissa Mulder-Hanekamp, Roy P. C. Kessels

**Affiliations:** 1 Department of Medical Psychology, Radboud University Medical Center, Internal Post 925, PO Box 9101, 6500 HB Nijmegen, The Netherlands; 2 Donders Institute for Brain, Cognition and Behaviour, Radboud University, Nijmegen, The Netherlands; 3 Division Diagnostics, Research, and Education, Forensic Psychiatric Center Pompestichting, Nijmegen, The Netherlands; 4 Department of Clinical Psychology, Leiden University, Leiden, The Netherlands; 5 Leiden Institute for Brain and Cognition, Leiden, The Netherlands; 6 School of Psychology, Radboud University, Nijmegen, The Netherlands; 7 Stumass, JADOS Foundation, Zwolle, The Netherlands; 8 Vincent Van Gogh Institute for Psychiatry, Venray, The Netherlands

**Keywords:** Cognitive aging, Memory, Spatial learning, Neuropsychology

## Abstract

**Background:**

Healthy aging is accompanied by a decline in learning ability and memory capacity. One widely-studied method to improve learning outcome is by reducing the occurrence of errors during learning (errorless learning; EL). However, there is also evidence that committing errors during learning (trial-and-error learning; TEL) may benefit memory performance. We argue that these inconsistent findings could be driven by a lack of control over the error frequency in traditional EL and TEL paradigms.

**Aim:**

This study employed a spatial learning task to study EL and TEL and to determine the impact of error frequency on memory recall in healthy older adults (OA; *N* = 68) and young adults (YA; *N* = 60).

**Method:**

Four groups of participants (YA-EL, YA-TEL, OA-EL, OA-TEL) were instructed to first place and memorize the locations of everyday objects in a chest of drawers presented on a computer screen, and in whom memory recall performance was later tested. In the TEL condition, the amount of errors made before the correct drawer was ‘found’ was predetermined, varying from 0 to 5. During the EL condition, every first attempt was correct (i.e., no errors were made).

**Results:**

We found better overall performance in YA compared to OA and a beneficial effect of EL in both age groups. However, the amount of errors committed during learning did not influence accuracy of memory recall.

**Conclusion:**

Our results indicate that elimination of errors during learning can benefit memory performance in both YA and OA compared to TEL.

## Introduction

Healthy aging is accompanied by cognitive alterations, in addition to physical and neurobiological changes [1]. For instance, cognitive processes such as processing speed, working memory, episodic memory and executive functions, are susceptible to aging-related decline, while other cognitive abilities like semantic memory and problem-solving are preserved or even improve over the life span [1–5]. Moreover, following high-level education early in life, midlife work complexity, and late-life active engagement in social, physical and mental activities may all have a protective effect on aging-related cognitive disorders [6]. Successful participation in later-life activities and optimal functioning in a rapidly changing daily life thus requires optimal and life-long learning.

One approach that may improve learning outcome in healthy older adults (OA) is errorless learning (EL). Core element of the EL approach is that the occurrence of errors is prevented as much as possible (or even fully eliminated), typically resulting in superior learning compared to trial and error learning (TEL) in cognitively unimpaired older adults [7, 8]. However, beneficial effects of TEL, in which errors during learning are not eliminated, have also been reported in OA [9]. In contrast, others did not find any differences in performance in OA between EL and TEL [but showed a superior effect after EL in healthy *young* adults (YA)] [10]. Based on these mixed findings in healthy YA and OA, it has been argued that age may not be the defining factor, but that the adverse or beneficial effect of errors depends on the type of information to be encoded and retrieved. Cyr and Anderson, for instance, suggested that both YA and OA would benefit from errors in conceptual learning, whereas EL is preferable in nonconceptual learning [11, 12]. To either benefit from or being hampered by errors made during learning may also rely on the ability to successfully distinguish between correct and erroneous responses (that is, error monitoring, part of cognitive control). Several studies found that error monitoring declines with increasing age, resulting in slowing responses and greater proportion of undetected errors compared to YA [13–15].

To date, very little is known about the impact that the number of errors committed (i.e., error frequency) during acquisition has in healthy YA and OA. The role of error frequency has often been overlooked in previous studies, as paradigms neither employed systematical manipulation nor analyzed error rates. Note that in most commonly used EL paradigms participants had to complete word stems or word pairs for which, in the EL condition, correct answers were immediately presented by the experimenter or, in the TEL condition, correct answers had to be guessed by the participants in two to four attempts, but that were eventually also provided by the experimenter. This lack of systematic manipulation or control complicates the interpretation of previous studies may be a driving force behind inconsistent results [13].

In the present study, we compared memory recall performance in healthy YA and OA after EL and TEL using a spatial learning task, in which the amount of errors (defined as incorrect responses) committed during the acquisition phase of TEL was carefully manipulated [16]. That is, participants made 0, 2, 3, 4, or 5 incorrect responses (i.e., errors) before their response was considered to be correct. Moreover, as remembering word pairs or completing word stems bears little resemblance to everyday life demands, we adopted a more ecologically valid approach, that is, by employing a visuospatial learning task in which participants had to search for objects at different locations and remember these for later use, processes that are also highly relevant for everyday functioning (e.g. enabling us to recall where we have stored our wallet, keys, or glasses) [17]. We hypothesized that YA would perform better after EL compared to TEL, but that error frequency would not influence recall performance in this group, thus replicating previous findings in YA [16]. In addition, Kessels et al. [10] and Ariel and Moffat [18] demonstrated that OA perform worse than YA on explicit spatial learning and memory tasks, for instance tasks in which participants had to acquire, store and retrieve the locations of everyday objects in one of five virtual rooms (living room, bedroom, study room, bathroom and kitchen), but that implicit spatial learning, metacognition and navigation were largely preserved at older age. Based on these findings and the finding that error monitoring is susceptible to aging-related decline [14, 15], thus adding to the effects of ageing-related episodic memory decline [1–4], we hypothesized that a higher amount of errors would interfere more with learning in OA than in YA, which should be reflected by a reduced recall accuracy in the OA specifically.

## Methods

### Participants

We used a between-subject design and recruited four groups of healthy adults: 30 OA (with an age range from 54 to 75) were assigned to a group that performed a TEL task; 30 OA to an EL group; 38 YA (with age ranging between 17 and 38) were included in a TEL group and 30 YA in an EL group.^1^ Recruitment took place at Radboud University, HAN University of Applied Science, and a hiking club for the elderly and in the Epe area of the Netherlands. Intelligence was estimated with either the Raven’s Advanced Progressive Matrices-Short Form [19] or the National Adult Reading Test [20]. Level of intelligence was defined as low (IQ < 85), average (IQ = 85–115) and high (IQ > 115). The OA were cognitively screened using the Mini-Mental State Examination [21], and those who scored below 24 were excluded from participation (see Table 1 for an overview of the demographic characteristics of the participants) [22]. The local ethics review committee of the Faculty of Social Sciences of Radboud University approved the study and written informed consent was provided by all participants. Participation was voluntary and no financial reimbursement was provided to the YA; the elderly participants received a €10 gift voucher for their participation.

**Table 1:** Demographics of the participants per group

	Young adults		Older adults	
	TEL (*N* = 38)	EL (*N* = 30)	TEL (*N* = 30)	EL (*N* = 30)
Sex, M/F	3/35	13/17	16/14	7/23
Mean age (SD)	21.7 (4.80)	21.1 (2.24)	63.5 (5.76)*	68.2 (5.45)*
Mean IQ	Average	Average	Average	Average
Mean MMSE (SD)			29.1 (1.30)	28.8 (1.86)

*The mean difference in age of the older adults between EL and TEL is statistically significant (*p* = 0.001)

### Materials

We used a recently developed computerized object-location learning and memory task (i.e., the Drawer task), that can be used to study both EL and TEL [16]. The task consisted of an acquisition round that was immediately followed by a free-recall round. A 5 × 5 chest of drawers was shown on a computer screen in both learning conditions, in which pictures of common, easy-to-name objects had to be either found or stored (depending on the task condition). There was no time limit.

**Fig. 1:**
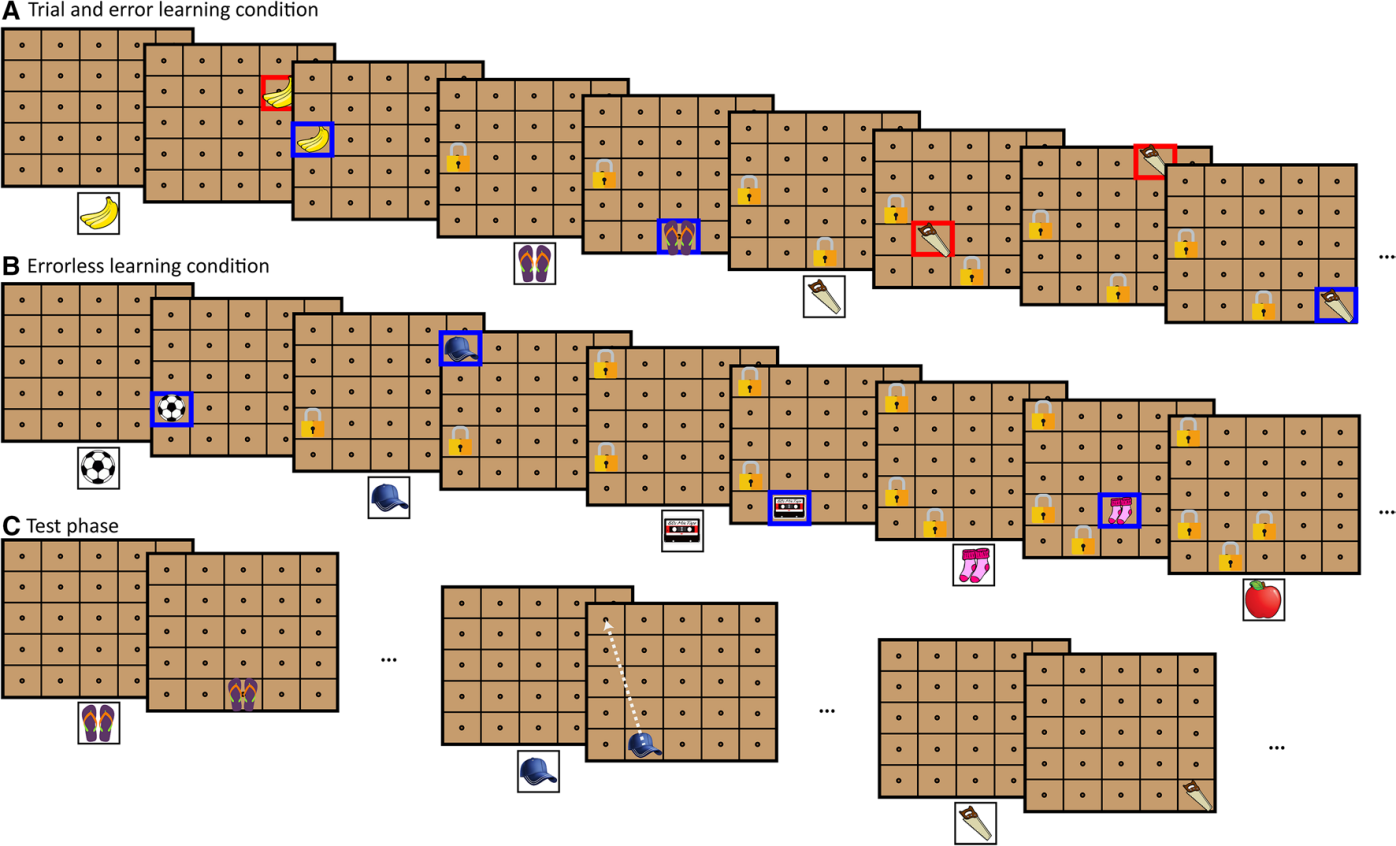
Schematic outline of the Drawer task. During the learning phase in the TEL condition (**a**), the participant must find a ‘hidden’ object (shown at the bottom of the screen) in one of the 25 drawers. A red square appears when the participant has chosen an incorrect drawer, after which the participant must select another drawer until the correct drawer was found. A blue square appears when the correct drawer is found followed by a lock, indicating that no other object is hidden in that drawer. The participant has to memorize the location of the object. Subsequently, the next object is shown at the bottom of the screen and the participant must find the correct drawer. In the EL condition (**b**), the participant has to place an object (shown at the bottom of the screen) in one of the 25 drawers. A blue square followed by a lock is shown, indicating that the chosen drawer is correct and that no other object can be placed in that drawer. The location of the selected drawer must be memorized for later recall and the next drawer is presented at the bottom of the screen. In the learning phase (**c**), the previously presented objects are shown at the bottom of the screen in random order and the participant has to indicate the drawer in which the object was previously stored (by placing the object in that drawer). No feedback was given about the accuracy of their choice. The distance score (i.e., the absolute distance between the target location and the recalled location) is indicated by the dashed arrow

During the TEL acquisition phase, participants were instructed to find 20 different objects, appearing at the bottom of the screen, in one of the 25 drawers by clicking on a drawer of their choice. A blue square appeared around the selected drawer when it was the correct choice, after which a lock appeared on the drawer, indicating that this drawer was unavailable for storing the remaining objects. A red square appeared around the selected drawer in case it was not the correct location, and participants had to continue their search for the correct drawer (see Fig. 1a). Participants were instructed to memorize the correct location of each object for later recall. Unknown to the participant, the amount of errors made (0, 2, 3, 4, or 5) before the correct drawer was identified during the acquisition phase was predetermined. Sometimes the first allocation of an object into a drawer was correct (i.e., an errorless item), whereas for other objects 2, 3, 4 or 5 errors had to be made before the correct drawer was found (i.e., trial-and-error items). There were four trials per error frequency manipulation and the order of the stimuli with a particular error frequency was random to prevent participants from anticipating the number of incorrect attempts.

During the EL condition, 20 objects were subsequently presented at the bottom of the screen, and participants had to randomly place each object into an available drawer and memorize its location for later recall. In this condition, the ‘correct’ drawer was defined as the participant’s first chosen drawer for each object, so that no errors were made during the acquisition (see Fig. 1b). Again, participants were instructed to memorize the location of each object for later recall.

In the recall phase (see Fig. 1c), which was identical for both task conditions, participants were shown the same drawer unit (with the locks removed) and the objects were presented serially underneath the drawer unit. The participants were instructed to place each object into the correct drawer that was ‘discovered’ in the acquisition phase without a time limit. They had only one attempt to place an object in the correct drawer and no feedback was given about the accuracy of their choice. The objects were presented in randomized order across the participants.

The performance of the participants on the Drawer Task was measured by the number of incorrectly recalled locations in the free-recall test across participants (with a maximum of 20 errors; referred to as the ‘error score’). A second measure was the absolute distance between the target location and the recalled location averaged across all 20 items (with a maximum of 5.66 in arbitrary units, referred to as the ‘distance score’, for more details see [16]).

### Data analysis

First, to examine whether YA and OA benefited from EL compared to TEL, two General Linear Model (GLM) analyses were performed with group (YA vs. OA) and learning condition (EL vs. TEL condition) as between-subject factors and, respectively, the error score and distance score as dependent variables.

For the TEL condition only, a repeated measures GLM was conducted with group (YA vs. OA) as between-subject factor, error frequency (performance on the 0, 2, 3, 4 and 5-error trials) as within-subject factor and the error score and distance score as dependent variables to examine the effects of error frequency during the acquisition phase on subsequent recall.

## Results

**Fig. 2:**
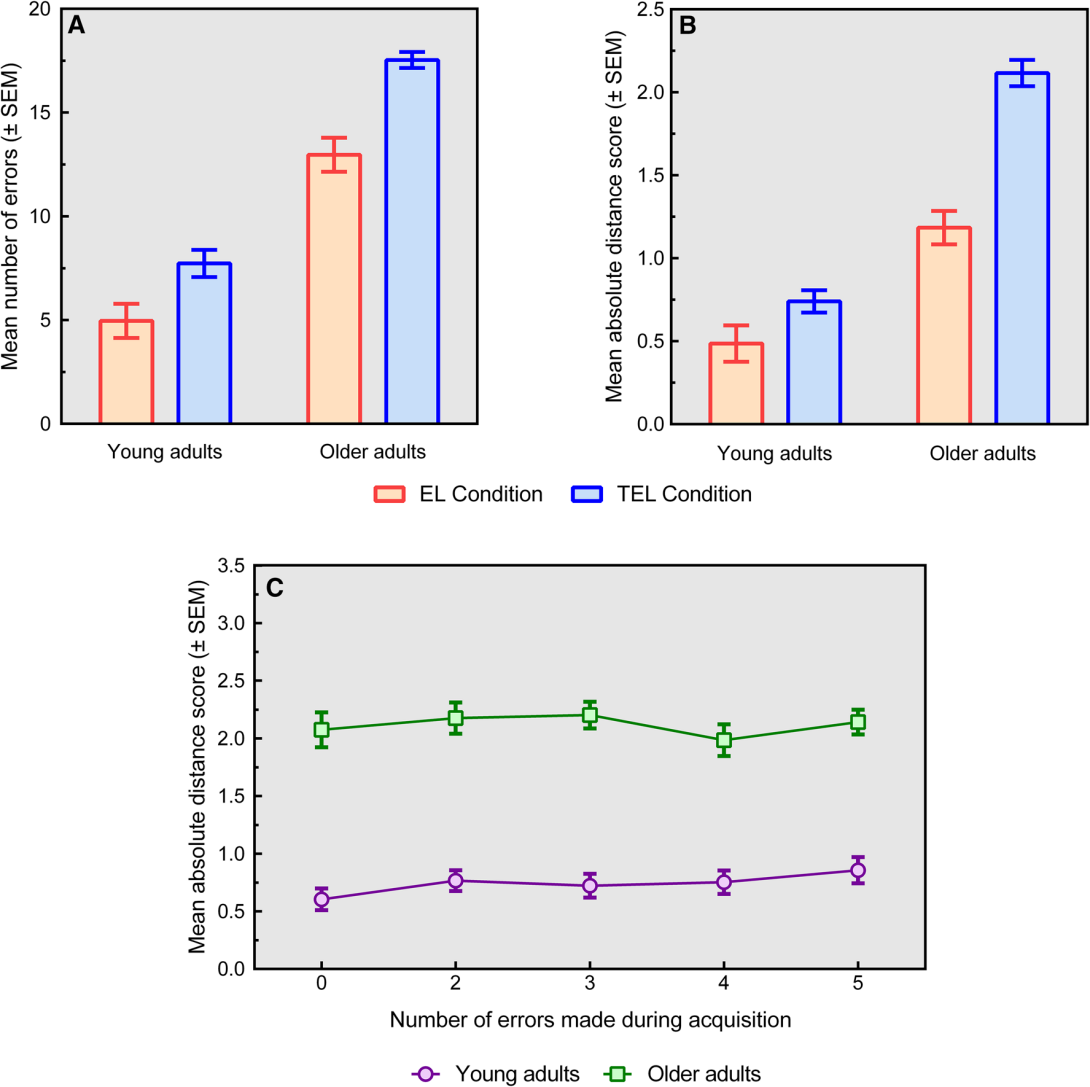
**a** Mean number of errors (± SEM) per group and condition. **b** Mean distance score (± SEM) per group and condition. **c** The distance score (± SEM) per learning condition of TEL (performance on the 0, 2, 3, 4 and 5 error trials) for young and older adults

Both groups performed significantly better after EL (YA: *M* = 4.97, SD = 4.51; OA: *M* = 13.0, SD = 4.48) than after TEL (YA: *M* = 7.74, SD = 4.04; OA: *M* = 17.5, SD = 2.11) and YA overall made significantly less errors than OA (Learning condition: *F*(1, 124) = 27.7, *p* < 0.001, *η*_p_^2^ = 0.183; Group: *F*(1, 124) = 163, *p* < 0.001, *η*_p_^2^ = 0.568). However, there was no interaction effect when examining the number of errors made (*F*(1, 124) = 1.66, *p* = 0.200, *η*_p_^2^ = 0.013, see also Fig. 2a).

The second GLM analysis also showed an EL advantage in both groups (YA: *M* = 0.486, SD = 0.602; OA: *M* = 1.18, SD = 0.551) compared to TEL (YA: *M* = 0.740, SD = 0.414; OA: *M* = 2.12, SD = 0.437), and YA overall selected drawers which were closer in absolute distance to the correct drawer compared to OA (Learning condition: *F*(1, 124) = 44.3, *p* < 0.001, *η*_p_^2^ = 0.263; Group: *F*(1, 125) = 135, *p* < 0.001, *η*_p_^2^ = 0.522). Moreover, we found a significant interaction effect (*F*(1, 125) = 14.4, *p* < 0.001, *η*_p_^2^ = 0.104),^2^ indicating that the performance of OA suffered slightly more from errors made during TEL-based acquisition relative to EL (*M*_TEL-EL_ = 0.940) compared to YA (*M*_TEL-EL_ = 0.254; see also Fig. 2b).^3^

The results of the repeated-measures GLM testing whether error frequency during the acquisition phase of the TEL condition affected the subsequent recall of both groups were neither significant for the number of errors (Error frequency: *F*(3.84, 253) = 1.49, *p* = 0.208, *η*_p_^2^ = 0.022; Error frequency × Group: *F*(3.84, 253) = 0.190, *p* = 0.938, *η*_p_^2^ = 0.003) nor the absolute distance (Error frequency: *F*(3.88, 256) = 1.00, *p* = 0.405, *η*_p_^2^ = 0.015; Error frequency × Group: *F*(3.88, 256) = 0.642, *p* = 0.628, *η*_p_^2^ = 0.010, see also Fig. 2c).^4^

## Discussion

The purpose of the present study was to examine the impact of error frequency on memory outcome in YA and OA. Our results indicated that, in general, YA had a better overall memory performance than OA. Also, an EL advantage over TEL was consistently found in both age groups. In addition, errors made during the acquisition phase of the TEL condition interfered slightly more with the subsequent recall of OA compared to YA, that is, YA selected drawers which were in absolute distance closer to the correct drawer compared to OA. However, the amount of errors committed during learning was unrelated to later memory recall.

The beneficial effect of EL and the finding that occurrence of errors during learning interfere more with memory performance of OA than YA are in line with the findings of Lubinsky, Rich and Anderson [8] and Guild and Anderson [7]. Lubinsky, Rich and Anderson [8] reported an EL advantage in a task in which word stems were presented and OA were either instructed to generate an answer, or did not have to generate an answer as the answer was provided by the experimenter. In their study, no errors could be made in the learning phase of EL, as participants immediately generated the correct answer or the correct answer was given by the experimenter, while in their TEL condition participants made three guesses or the experimenter gave three incorrect answers before the correct answer was given that participants had to remember. Guild and Anderson [7] used a similar task and procedure, but in their study OA had to learn a semantically related or unrelated word list. They showed that, in OA, free recall was better after EL for both semantically related and unrelated word lists and for both self-generated and experimenter provided answers compared to TEL. The manipulations in these previous studies are similar to those in our paradigm (i.e., self-generated errors or immediately correct responses), although our paradigm removes the involvement of the experimenter during task performance, is visuospatial in nature, and may have a better ecological validity (as finding and remembering the locations of objects is relevant in daily life).

With respect to the underlying neurocognitive processes of the EL benefit, studies have found aging-related deficits in suppressing irrelevant information alongside relevant information. Consequently, irrelevant information is more likely to be encoded by OA than YA, resulting in worse recall performance [23–26]. Errors made during acquisition of TEL, i.e., irrelevant information, may have distracted and reduced the processing resources for the subsequent (relevant or irrelevant) item in OA to a greater extent than in YA, and subsequently conflicted with the correct response during retrieval of information resulting in worse overall recall performance and, when making an error, greater distance scores relative to YA. However, contrary to our hypothesis that the number of errors made would increasingly interfere with the learning outcome (especially in OA), we found that learning was influenced by error frequency in neither age group. One explanation could be that when the likelihood that errors will occur is relatively high, the value of each single error is reduced, as the occurrence of these errors are expected [27, 28]. Also, Ferdinand [29] demonstrated that OA learned worse when the value of negative feedback was reduced and rely more on positive than negative feedback, relative to YA. In line with these findings, our results indicate that OA employ a different strategy than YA by relying more on rewarding, positive feedback following correct responses irrespective of the error frequency, whereas the performance of YA could predominantly be influenced by the value of the first error [29]. The present study extends the previous results obtained using the Drawer task in cognitively unimpaired OA by showing that the same pattern of results (i.e., a beneficial effect of EL compared to TEL, but no effect of error frequency on memory outcome) can also be observed in YA [16].

In sum, we found that error elimination during learning benefited later recall of object locations compared to TEL in both YA and OA. The number of errors made, however, did not affect the performance in cognitively unimpaired YA and OA. Possibly, the impact of error frequency on learning outcome has a more profound influence on memory performance in patients with cognitive impairments, as suggested by Baddeley and Wilson [30]. Future research should explore this assumption in patients with impaired episodic memory and/or deficits in the error-monitoring system, such as older individuals with subjective cognitive complaints at risk for further decline to help them optimize their memory [31], or patients with mild cognitive impairment or dementia, in whom the underlying mechanisms of EL have been scarcely studied [32, 33]. Our experimental task, in which errors are systematically controlled for, may help to gain a better understanding of the underlying cognitive mechanisms of the learning capacity in cognitively impaired individuals with possible consequences for cognitive rehabilitation.

## Footnotes

1 The YA in the EL condition also participated in the study of Scheper et al. [16].

2 Note that separate univariate ANOVAs on the distance score with respectively YA or OA and learning condition as between-subject factor and EL or TEL and group as between-subject factor revealed significant effects (YA: learning condition: *F*(1, 66) = 4.26, *p* = 0.043, *η*_p_^2^ = 0.061; OA: learning condition: *F*(1, 58) = 52.6, *p* < 0.001, *η*_p_^2^ = 0.476; EL: group: *F*(1, 58) = 22.0, *p* < 0.001, *η*_p_^2^ = 0.275; TEL: group: *F*(1, 66) = 176, *p* < 0.001, *η*_p_^2^ = 0.728).

3 To examine the potential effect of the small, yet significant age difference between the two older groups, we performed two separate GLM analyses on the older adults only with learning condition (EL vs. TEL) as between-subject factor and, respectively, error score and distance score as dependent variable, adding age as covariate. This did not alter the initial results: a significant main effect of learning condition was still present (Error score: *F*(1, 57) = 35.1, *p* < 0.001, *η*_p_^2^ = 0.381; distance score: *F*(1, 57) = 74.6, *p* < 0.001, *η*_p_^2^ = 0.567).

4 We also explored whether the performance on the EL condition and the performance on the zero-error (‘errorless’) items of the TEL condition for the two groups (YA and OA) were different by calculating the percentage of the number of errors of the “errorless” trails of both conditions, since the EL condition consisted of 20 zero-error trials and the TEL condition of 4 zero-error trials. Next, two GLM analyses were conducted with these variables and showed a better overall performance of YA compared to OA and both groups performed significantly better after EL than TEL (Group: percentage of error score: *F*(1, 124) = 115, *p* < 0.001, *η*_p_^2^ = 0.480; distance score: *F*(1, 124) = 89.2, *p* < 0.001, *η*_p_^2^ = 0.418; Learning condition: percentage of error score: *F*(1, 124) = 10.8, *p* = 0.001, *η*_p_^2^ = 0.080; distance score: *F*(1, 124) = 19.3, *p* < 0.001, *η*_p_^2^ = 0.134). Also, a significant interaction effect of learning condition and group was demonstrated. Errors made during acquisition during TEL interfered more with the “errorless” items in OA than YA, resulting in a larger absolute distance (*F*(1, 124) = 11.3, *p* = 0.001, *η*_p_^2^ = 0.083), but not in a higher percentage of errors made (*F*(1, 124) = 1.98, *p* = 0.162, *η*_p_^2^ = 0.016).
